# Association between dietary approaches to stop hypertension eating pattern and lung cancer risk in 98,459 participants: results from a large prospective study

**DOI:** 10.3389/fnut.2023.1142067

**Published:** 2023-05-15

**Authors:** Zhiyong Zhu, Linglong Peng, Haitao Gu, Yunhao Tang, Yi Xiao, Hongmei He, Mingying Yang, Ling Xiang, Yaxu Wang

**Affiliations:** ^1^Department of Gastrointestinal surgery, The Second Affiliated Hospital of Chongqing Medical University, Chongqing, China; ^2^Department of Surgery Anesthesiology, The Second Affiliated Hospital of Chongqing Medical University, Chongqing, China; ^3^Department of Clinical Nutrition, The Second Affiliated Hospital of Chongqing Medical University, Chongqing, China

**Keywords:** DASH diet, lung cancer, PLCO trial, prevention, Cox regression analysis

## Abstract

**Background:**

Dietary approaches to stop hypertension (DASH) eating pattern is linked to anti-inflammatory responses and antioxidation, which overlap with the pathogenesis of lung cancer. However, there is insufficient epidemiological evidence to link this dietary pattern to lung cancer risk conclusively.

**Aim:**

To determine if adherence to the DASH diet is linked to a lower risk of developing lung cancer in a large prospective study.

**Methodology:**

The data of participants were retrieved from the Prostate, Lung, Colorectal, and Ovarian (PLCO) Cancer Screening Trial. A DASH score was calculated based on 8 dietary components to reflect adherence to DASH, with greater scores representing higher adherence. Three Cox proportional hazards models were constructed to analyze the association between DASH scores and lung cancer risk, including an unadjusted model and two adjusted models (model 1 for demographics and model 2 for fully confounding factors). A restricted cubic spline plot was utilized to illustrate the likelihood of developing lung cancer across the entire range of DASH scores. The association between each of the 8 DASH components and the risk of lung cancer was assessed separately. Several subgroup analyses were conducted to identify potential modifiers, and several sensitivity analyses were performed to verify the robustness of the findings.

**Results:**

The study involved 98,459 individuals in total. The mean (standard deviation) DASH score was 24.00 (4.62) points, along with the mean follow-up period of 8.84 (1.94) years. Lung cancer was identified in 1642 cases over 869807.9 person-years of follow-up, and the overall incidence rate was 0.189 cases/100 person-years. Participants in the highest quartile in the fully adjusted model had a relatively decreased risk of developing lung cancer in comparison to those in the lowest quartile (HR_quartile 4 versus 1_: 0.647; 95% CI: 0.557, 0.752; *P*_trend_ < 0.001). The restricted cubic spline plot demonstrated that DASH score and lung cancer risk were inversely associated and had a linear dose–response relationship (*P*_non-linear_ = 0.944). According to subgroup analyses, those who were current or former smokers had a stronger inverse connection than those who never smoked (*P*_interaction_ = 0.013). The results remained robust after several sensitivity analyses.

**Conclusion:**

The risk of lung cancer was inversely associated with DASH scores in the US population. This suggests that following the DASH pattern can help prevent lung cancer, especially for current or former smokers. More epidemiological evidence from other regions and populations is needed to confirm our findings.

## Introduction

Lung cancer is the second most widely occurring cancer, with about 2.24 million new cases recorded globally in 2020. It has contributed to 18% of all cancer fatalities (1). For a long time, smoking has been considered a key risk factor for lung cancer, however, an increasing number of people who have never smoked are being diagnosed with lung cancer (2). Therefore, the increasing rate of lung cancer cannot be attributed to smoking alone. In addition to smoking status, duration and intensity, other factors may also play a role in the pathogenesis of lung cancer, such as heredity (3), alcohol consumption (4), air pollution (5) and diet (6). Specially, numerous studies have identified various dietary components as a potential modifiable risk factor for lung cancer. For example, a prospective cohort study from the United Kingdom showed that adding fruits, vegetables, and dietary fiber to the diet was related to lower lung cancer risk, but high consumption of processed and red meat elevated the risk of developing the disease (7). Another observational study also showed that high consumption of sodium was associated with an increased risk of developing lung cancer (8).

Nowadays, research on dietary patterns rather than single foods or nutrients is being sought after for its improved science (9). Dietary approaches to stop hypertension (DASH) eating pattern was established from a hypertension control program in the USA (10). It encourages people to consume more fruits, vegetables, nuts and legumes, low-fat dairy products, and whole grains and to consume less sodium, beverages with high sugar content, processed and red meat. Obviously, certain dietary components in the DASH dietary pattern, such as fruits, vegetables, sodium, processed and red meat are closely associated with the occurrence of lung cancer. In addition, although the DASH pattern was originally designed for the prevention and control of hypertension, studies have revealed that it is also linked to anti-inflammatory responses and anti-oxidative damage, which overlap with the pathogenesis of lung cancer (11, 12).

Based on the above, a hypothesis was made that there might be a correlation between DASH dietary pattern and the likelihood of developing lung cancer. In order to provide epidemiological evidence for the possible association, we performed this prospective designed analysis in a large US population.

## Methodology

### Study design and population

**Figure 1:**
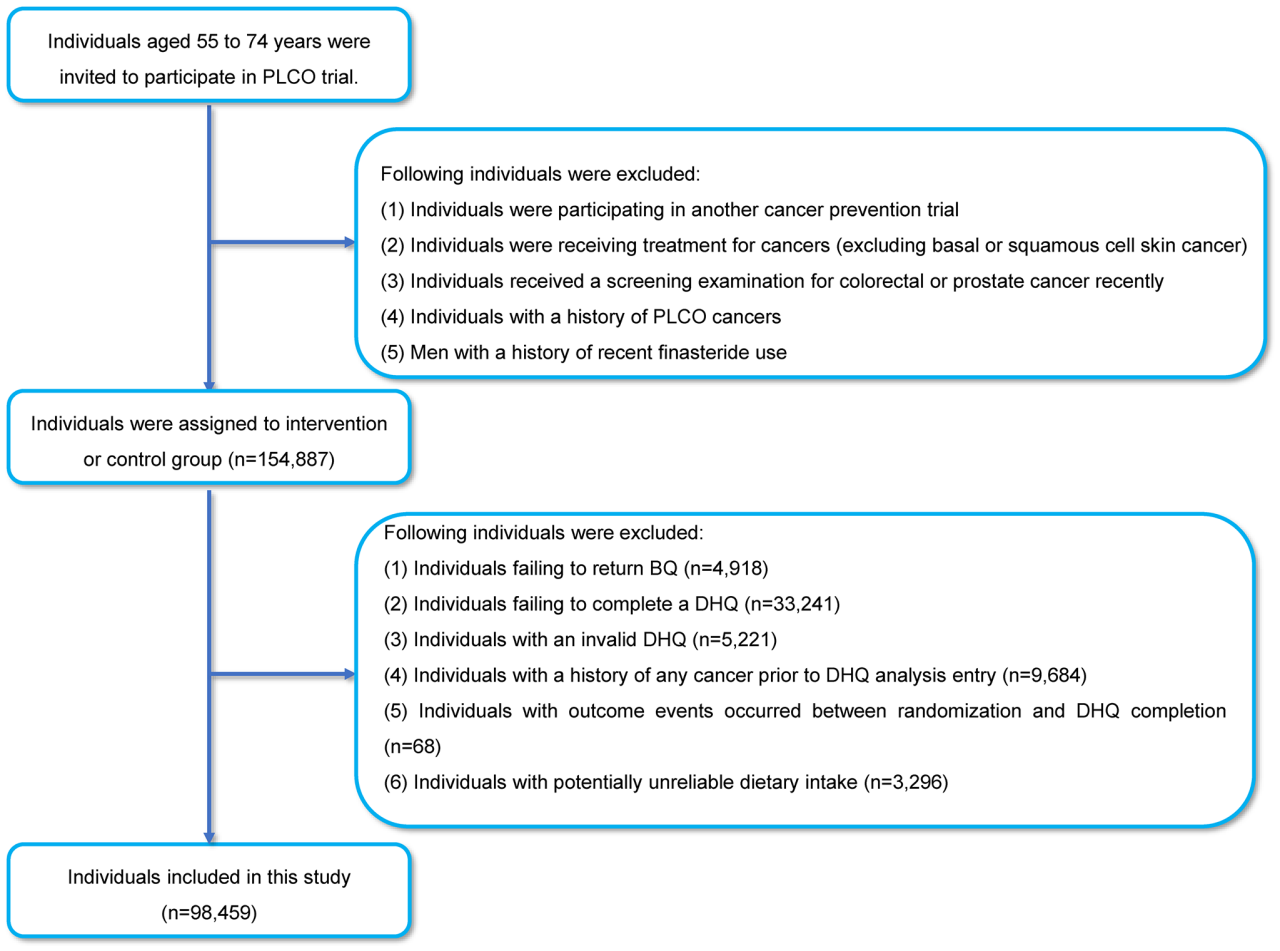
The flow chart of identifying participants included in our study.

**Figure 2:**
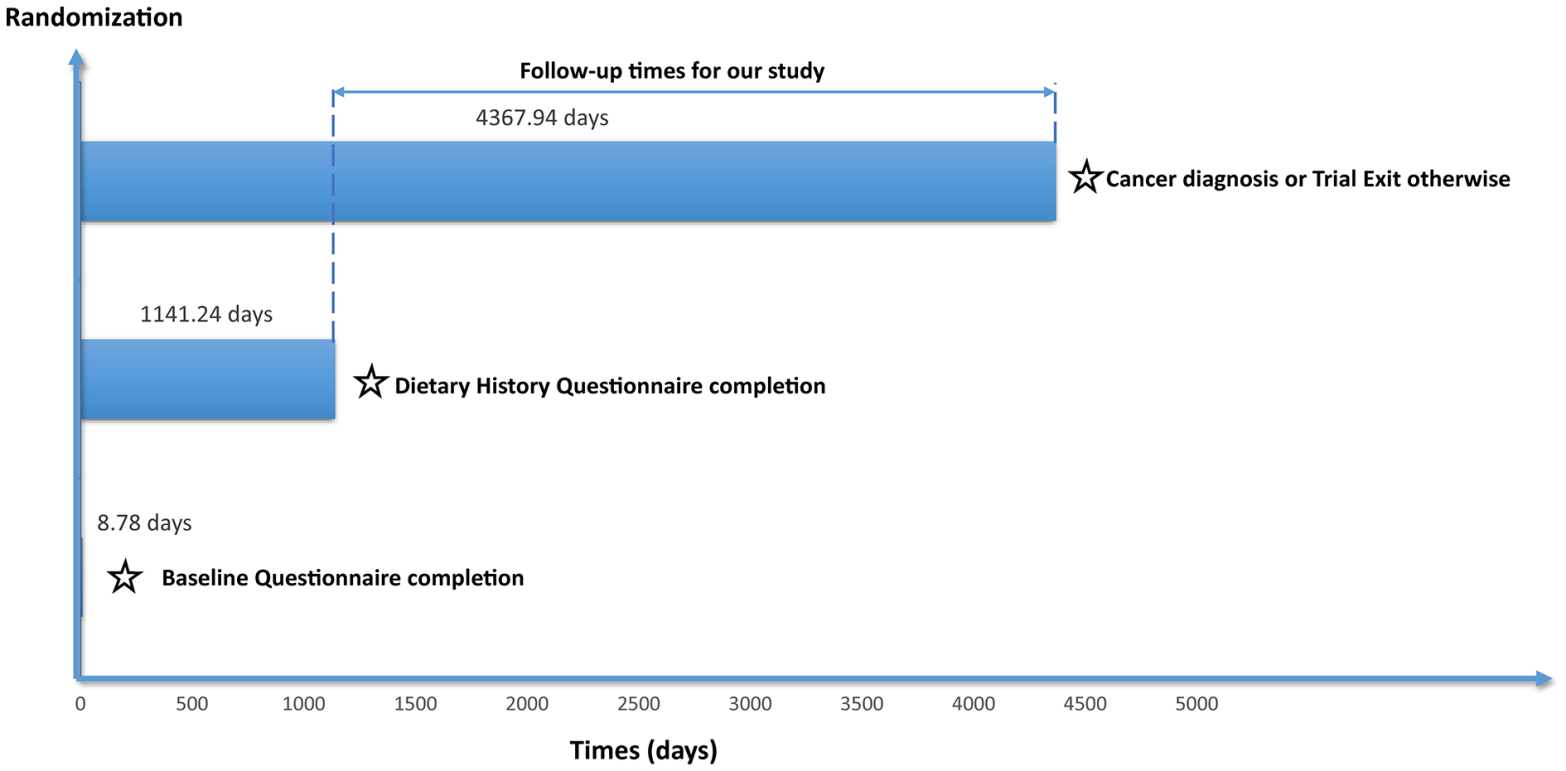
The timeline and follow-up scheme of our study.

All data used in our study was obtained from the Prostate, Lung, Colorectal, and Ovarian (PLCO) Cancer Screening Trial, a randomized, controlled study funded by the National Cancer Institute (NCI) aimed at assessing whether specific screening tests could reduce mortality resulting from PLCO cancers. Study design of the PLCO trial has been provided in the initial literature (13). The trial enrolled 154,887 participants between November 1993 and July 2001, aged 55–74 years from ten different centers, after obtaining written informed consent from each participant and meeting the eligibility inclusion criteria (Figure 1). Participants were each randomly assigned to the control or the intervention group in equal proportions. Regular care was provided to participants assigned to the control group, whereas those assigned to the intervention group received predefined screening exams for PLCO cancers. In PLCO trial, each participant was asked to complete several questionnaires, including the baseline questionnaire (BQ) and diet history questionnaire (DHQ), based on his or her real-life condition. BQ was utilized to gather data such as demographic features that participants could actively report as baseline information. The DHQ was a food frequency questionnaire that asked about the quantity and frequency of diet intake during the previous year (14, 15). The raw responses collected from participants were processed into analysis-ready variables. Simply put, the frequency of receiving a particular item was multiplied by the number of servings taken each day to estimate the daily consumption with the aid of the DietCalc software (16). DHQ is a frequently used nutritional assessment scale, and its validity has been confirmed elsewhere (17). The information on cancer diagnoses until 2009 and the patients’ mortality until 2018 were also collected. In our present study, as we aimed to investigate the association between DASH and lung cancer risk, the follow-up time was defined as the interval between the date of completion of the DHQ and the occurrence of lung cancer, death, loss during follow-up, or the end of the follow-up period (i.e., December 31, 2009), whichever event occurred first (Figure 2).

According to our study design, participants who met the following criteria were further excluded (Figure 1): (i) participants who failed to complete the BQ (n = 4,918); (ii) participants who failed to complete the DHQ (n = 33,241); (iii) participants who completed the invalid DHQ, such as missing ≥8 frequency responses (n = 5,221); (iv) participants who had any cancer diagnosis in their history before DHQ entry (n = 9,684); (v) participants who had lung cancer between DHQ entry and DHQ completion (n = 68); (vi) individuals who had potentially unlikely extreme energy intake (n = 3,296). Extreme energy intake in our study was classified as <800 kcal/day or > 4,200 kcal/day for men and < 600 kcal/day or > 3,500 kcal/day for women (18). Specially, our present study was conducted based on the data obtained with permission from the NCI (approval number: PLCO-1137).

### Assessment of DASH score

A DASH score was employed to describe the adherence of participants to the DASH pattern. The calculation of DASH score was proposed by Dr. Fung et al. in 2008 (19). For each participant, the intake of each DASH component was collected from the DHQ mentioned above. Based on the consumption data of each component, participants were divided into quintiles. In the case of vegetables, fruits, low-fat dairy products, nuts and legumes, and whole grains, participants in the lowest quintile scored 1 point, whereas those in the highest quintile scored 5 points. However, in the case of sodium, beverages with high sugar content, red and processed meat, participants in the lowest quintile scored 5 points, and those in the highest quintile scored 1 point. Criteria for determining scores are shown in Supplementary Table 1. The final DASH score was recorded by summing the scores of each component with a range of 8–40. As a result, the participants were more adherent to the DASH diet, as indicated by a higher score.

### Determination of lung cancer

Each participant in the study received an annual report *via* mail that included a form that asked them to indicate if they were diagnosed with cancer, the date of that diagnosis, and the location of the malignancy. Participants were contacted again by phone or email if they did not respond to the form. Death certificates and family reports were also viewed as sources of information. In addition, for participants who were diagnosed with lung cancer, their medical records were reviewed for confirmation of the lung cancer diagnosis and more details about this cancer.

### Assessment of covariates

The BQ was used in this research to gather data on gender, randomization group, smoking status, numbers of cigarettes smoked per day during smoking, number of packs smoked per day multiplied by years smoked (pack-years), race, hypertension history, and family history of lung cancer. Body mass index (BMI) was measured as weight (kg)/height square (m^2^). The DHQ was used to determine age, daily calorie intake from diet (kcal), and alcohol consumption profile.

### Statistical analysis

Missing data for categorical (family history of lung cancer, race, smoking status, cigarettes smoked per day, history of hypertension) and continuous variables (BMI, pack-years) with missing values <5% were imputed by the modal value and median, respectively, (20). The data characteristics before and after imputation are demonstrated in Supplementary Table 2. Subsequent analysis was carried out based on the complete data set after imputation.

To describe the link between DASH scores and lung cancer risk, the Cox proportional risk model was used, with follow-up time as the time metric. In order to illustrate this relationship, hazard ratios (HRs) and 95% confidence intervals (CIs) were calculated. In this model, all individuals were classified into quartiles as per their DASH scores, and the person-years of each quartile were determined independently. The reference group served as the first quartile with the lowest score. HRs and 95% CIs for the other quartiles were calculated and compared with the first quartile. Model 1 was adjusted for age, gender, and race, whereas model 2 was additionally adjusted for drinking status, smoking status, cigarettes smoked per day, pack-years, BMI, randomization group, hypertension history, family history of lung cancer, and dietary energy intake in multivariate analysis. The adjusted covariates in the above two models were selected based on existing literature rather than our subjective tendency to make conclusions statistically significant (6). Small cell carcinoma and non-small cell carcinoma risk were investigated separately to further investigate the relationship between the risk of subtypes of lung cancer and DASH scores. The median score for individual quartiles was allocated to all participants in the group and used as a continuous variable to understand the overall trend in lung cancer risk between groups. The *p*_value_ for the trend test was calculated to roughly describe the convergent relationship between DASH scores and risk for developing lung cancer. A restricted cubic spline plot with three knots at the 10th, 50th, and 90th percentiles was utilized to demonstrate the likelihood of developing lung cancer over the full range of DASH scores. *P*_non-linear_ was calculated to elucidate whether lung cancer risk and DASH scores have a non-linear dose–response relationship. In addition, by treating the intake of individual components as continuous variables, the link between the consumption of each component and the likelihood of lung cancer was investigated separately to help us identify the potential contributors to the association between DASH pattern and the risk of developing lung cancer.

After classification for age (≤65 vs. >65 years), gender (male vs. female), race (white vs. colored), randomization group (intervention group vs. control group), BMI (≤25 vs. >25 kg/m^2^), smoking status (never-smokers vs. current/former smokers), drinking status (no vs. yes), family history of lung cancer (no vs. yes/possible), history of hypertension (no vs. yes) and energy intake from diet (≤ median vs. > median), a series of prespecified subgroup analyses were carried out. *p*_value_ for likelihood ratio tests were computed to find out the significance of the interaction. Several sensitivity analyses were conducted to verify the robustness of the findings: (i) excluded participants with missing information to avoid the effect of data imputation on the final outcomes; (ii) eliminated individuals having a family history of lung cancer, as they might be more susceptible to developing lung cancer; (iii) excluded individuals who had a history of hypertension, since they might follow DASH-like dietary patterns to manage their condition; (iv) eliminated cases identified within the first two and four years of follow-up for ruling out any reverse causality.

R software 4.2.1 was utilized for statistical analyses. A two-tailed *p*_value_ of 0.05 was utilized to assess if the outcomes were statistically significant.

## Results

### Baseline characteristics

The mean value (standard deviation) of the DASH score for the 98,459 individuals in the current study was 24.00 (4.62) points. All individuals were classified into quartiles according to DASH scores as follows: quartile 1, DASH score 8–21, n = 29,523; quartile 2, DASH score 22–24, n = 23,433; quartile 3, DASH score 25–27, n = 22,564; quartile 4, DASH score 28–40, n = 22,939. The baseline features of the individuals are displayed in Table 1 by quartile. Subjects in the top quartile were more likely to be female, never-smokers, never-drinkers and had less tobacco exposure, a lower BMI and a history of hypertension in contrast with those in the lowest quartile. Individuals in the highest quartile of the DASH score consumed more fruits, nuts and legumes, vegetables, whole grains, low-fat dairy products and less sodium, sugared beverages, red and processed meats than those in the lowest quartile.

**Table 1:** Baseline characteristics of study population according to quartiles of DASH scores. Values are means (standard deviation) for continuous variables and percentages for categorical variables.

		Quartiles of DASH scores			
Characteristics	Overall	Quartile 1 (8–21)	Quartile 2 (22–24)	Quartile 3 (25–27)	Quartile 4 (28–40)
Number of participants	98,459	29,523	23,433	22,564	22,939
DASH score	24.00 ± 4.62	18.56 ± 2.28	23.03 ± 0.81	25.95 ± 0.82	30.08 ± 1.96
Age	65.52 ± 5.73	64.51 ± 5.57	65.51 ± 5.71	65.98 ± 5.69	66.38 ± 5.79
**Gender**					
Male	47,218 (47.96%)	18,320 (62.05%)	11,607 (49.53%)	9,574 (42.43%)	7,717 (33.64%)
Female	51,241 (52.04%)	11,203 (37.95%)	11,826 (50.47%)	12,990 (57.57%)	15,222 (66.36%)
**Race**					
White	91,221 (92.65%)	27,219 (92.20%)	21,774 (92.92%)	21,069 (93.37%)	21,159 (92.24%)
Non-white	7,238 (7.35%)	2,304 (7.80%)	1,659 (7.08%)	1,495 (6.63%)	1780 (7.76%)
Body mass index (kg/m^2^)	27.20 ± 4.79	27.95 ± 4.81	27.45 ± 4.78	27.05 ± 4.73	26.14 ± 4.61
**Family history of lung cancer**					
No	85,845 (87.19%)	25,429 (86.13%)	20,523 (87.58%)	19,733 (87.45%)	20,160 (87.89%)
Yes/ Possible	12,614 (12.81%)	4,094 (13.87%)	2,910 (12.42%)	2,831 (12.55%)	2,779 (12.11%)
**Smoker**					
Never	47,233 (47.97%)	11,561 (39.16%)	10,937 (46.67%)	11,708 (51.89%)	13,027 (56.79%)
Current/ Former	51,226 (52.03%)	17,962 (60.84%)	12,496 (53.33%)	10,856 (48.11%)	9,912 (43.21%)
Pack-years^a^	17.49 ± 26.40	23.99 ± 30.46	17.89 ± 26.33	14.57 ± 23.66	11.60 ± 20.99
**Cigarettes smoked per day**					
0	47,233 (47.97%)	11,561 (39.16%)	10,937 (46.67%)	11,708 (51.89%)	13,027 (56.79%)
1–20	32,197 (32.70%)	10,098 (34.20%)	7,919 (33.80%)	7,211 (31.96%)	6,969 (30.38%)
>20	19,029 (19.33%)	7,864 (26.64%)	4,577 (19.53%)	3,645 (16.15%)	2,943 (12.83%)
**Randomization group**					
Intervention group	50,151 (50.94%)	15,024 (50.89%)	11,828 (50.48%)	11,575 (51.30%)	11,724 (51.11%)
Control group	48,308 (49.06%)	14,499 (49.11%)	11,605 (49.52%)	10,989 (48.70%)	11,215 (48.89%)
**Drinker**					
No	26,681 (27.10%)	7,457 (25.26%)	6,114 (26.09%)	6,130 (27.17%)	6,980 (30.43%)
Yes	71,778 (72.90%)	22,066 (74.74%)	17,319 (73.91%)	16,434 (72.83%)	15,959 (69.57%)
**History of hypertension**					
No	66,641 (67.68%)	19,587 (66.34%)	15,651 (66.79%)	15,250 (67.59%)	16,153 (70.42%)
Yes	31,818 (32.32%)	9,936 (33.66%)	7,782 (33.21%)	7,314 (32.41%)	6,786 (29.58%)
Energy intake from diet (kcal/day)	1728.71 ± 658.04	1792.50 ± 685.67	1709.94 ± 679.96	1704.04 ± 658.02	1690.05 ± 589.63
**DASH components intake**					
Fruits (g/day)	275.24 ± 213.29	172.80 ± 161.71	254.35 ± 194.61	310.29 ± 207.58	393.94 ± 226.47
Nuts and legumes (g/day)	20.57 ± 26.06	12.08 ± 15.84	17.42 ± 20.40	21.89 ± 24.46	33.40 ± 36.18
Vegetables (g/day)	284.83 ± 181.87	210.68 ± 138.99	261.72 ± 159.58	304.33 ± 176.62	384.70 ± 206.03
Grains (g/day)	61.53 ± 59.68	33.96 ± 38.55	54.11 ± 50.11	70.08 ± 59.14	97.17 ± 71.17
Low-fat dairy (g/day)	137.18 ± 222.00	47.98 ± 131.73	108.89 ± 198.87	170.04 ± 235.35	248.57 ± 264.19
Sodium from diet (mg/day)	2728.47 ± 1126.48	2788.05 ± 1135.77	2708.86 ± 1166.99	2712.09 ± 1148.16	2687.92 ± 1044.88
Sugared beverages (g/day)	398.08 ± 463.51	535.76 ± 578.37	401.38 ± 442.44	344.63 ± 389.73	270.07 ± 314.11
Red/processed meats (g/day)	12.26 ± 14.62	19.59 ± 18.49	12.86 ± 13.24	9.48 ± 11.18	4.96 ± 6.72

a The product of the daily cigarette pack consumption and the number of years of smoking.

### Association between DASH scores and lung cancer risk

In this study, the mean (standard deviation) of the follow-up period was 8.84 (1.94 years) years. In total, 1,642 cases of lung cancer, comprising 234 small cell carcinoma and 1,408 non-small cell carcinoma cases, were recorded during 869807.9 person-years. The overall incidence was 0.189 cases per 100 person-years. In the unadjusted model, individuals in the highest quartile had a considerably decreased risk of lung cancer than the ones in the lowest quartile, as shown in Table 2 (HR_quartile 4 versus 1_: 0.470; 95% CI: 0.407, 0.543; *P*_trend_ < 0.001), the inverse relationship was still noted in the fully adjusted model (HR_quartile 4 versus 1_: 0.647; 95% CI: 0.557, 0.752; *P*_trend_ < 0.001). Moreover, when the observed lung cancer cases were analyzed separately for small cell and non-small cell carcinoma, the inverse relationship persisted after adjustment for possible confounders (For small cell carcinoma, HR_quartile 4 versus 1_: 0.363; 95% CI: 0.224, 0.588; *P*_trend_ < 0.001; For non-small cell carcinoma, HR_quartile 4 versus 1_: 0.693; 95% CI: 0.591, 0.812; *P*_trend_ < 0.001).

**Table 2:** Association of DASH scores with the risk of lung cancer and its subtypes.

Quartiles of DASH score	No. ofparticipants	No. ofcases	person-years	Hazard ratio (95% confidence interval)		
				Unadjusted	Model 1^a^	Model 2^b^
**Overall**						
Quartile 1 (8–21)	29,523	669	256327.5	1.00 (reference)	1.00 (reference)	1.00 (reference)
Quartile 2 (22–24)	23,433	387	206820.1	0.715 (0.631,0.811)	0.687 (0.606,0.779)	0.826 (0.728,0.937)
Quartile 3 (25–27)	22,564	331	199987.0	0.632 (0.554,0.721)	0.599 (0.524,0.685)	0.805 (0.704,0.921)
Quartile 4 (28–40)	22,939	255	206673.4	0.470 (0.407,0.543)	0.445 (0.384,0.516)	0.647 (0.557,0.752)
*P* for trend				<0.001	<0.001	<0.001
**Small cell carcinoma**						
Quartile 1 (8–21)	29,078	104	254480.7	1.00 (reference)	1.000 (reference)	1.00 (reference)
Quartile 2 (22–24)	23,138	63	205412.8	0.749 (0.548,1.024)	0.711 (0.518,0.974)	0.876 (0.638,1.202)
Quartile 3 (25–27)	22,230	46	198154.7	0.566 (0.400,0.801)	0.526 (0.370,0.749)	0.736 (0.516,1.049)
Quartile 4 (28–40)	22,605	21	204617.8	0.250 (0.156,0.399)	0.231 (0.143,0.372)	0.363 (0.224,0.588)
*P* for trend				<0.001	<0.001	<0.001
**Non-small cell carcinoma**						
Quartile 1 (8–21)	29,449	567	256066.4	1.00 (reference)	1.00 (reference)	1.00 (reference)
Quartile 2 (22–24)	23,380	323	206596.1	0.704 (0.614,0.807)	0.678 (0.591,0.778)	0.811 (0.707,0.932)
Quartile 3 (25–27)	22,517	284	199745.2	0.640 (0.555,0.738)	0.609 (0.527,0.704)	0.811 (0.701,0.938)
Quartile 4 (28–40)	22,879	234	206254.2	0.510 (0.438,0.594)	0.484 (0.414,0.567)	0.693 (0.591,0.812)
*P* for trend				<0.001	<0.001	<0.001

a Adjusted for age (years), gender (male, female) and race (white, non-white).

b Adjusted for model 1 plus drinking status (no, yes), smoking status (never, current/former), cigarettes smoked per day (0, 1–20, >20), pack-years (continuous), body mass index (continuous), randomization group (intervention group/control group), history of hypertension (no, yes), family history of lung cancer (no, yes/possible) and energy intake from diet (continuous).

### Additional analyses

**Figure 3:**
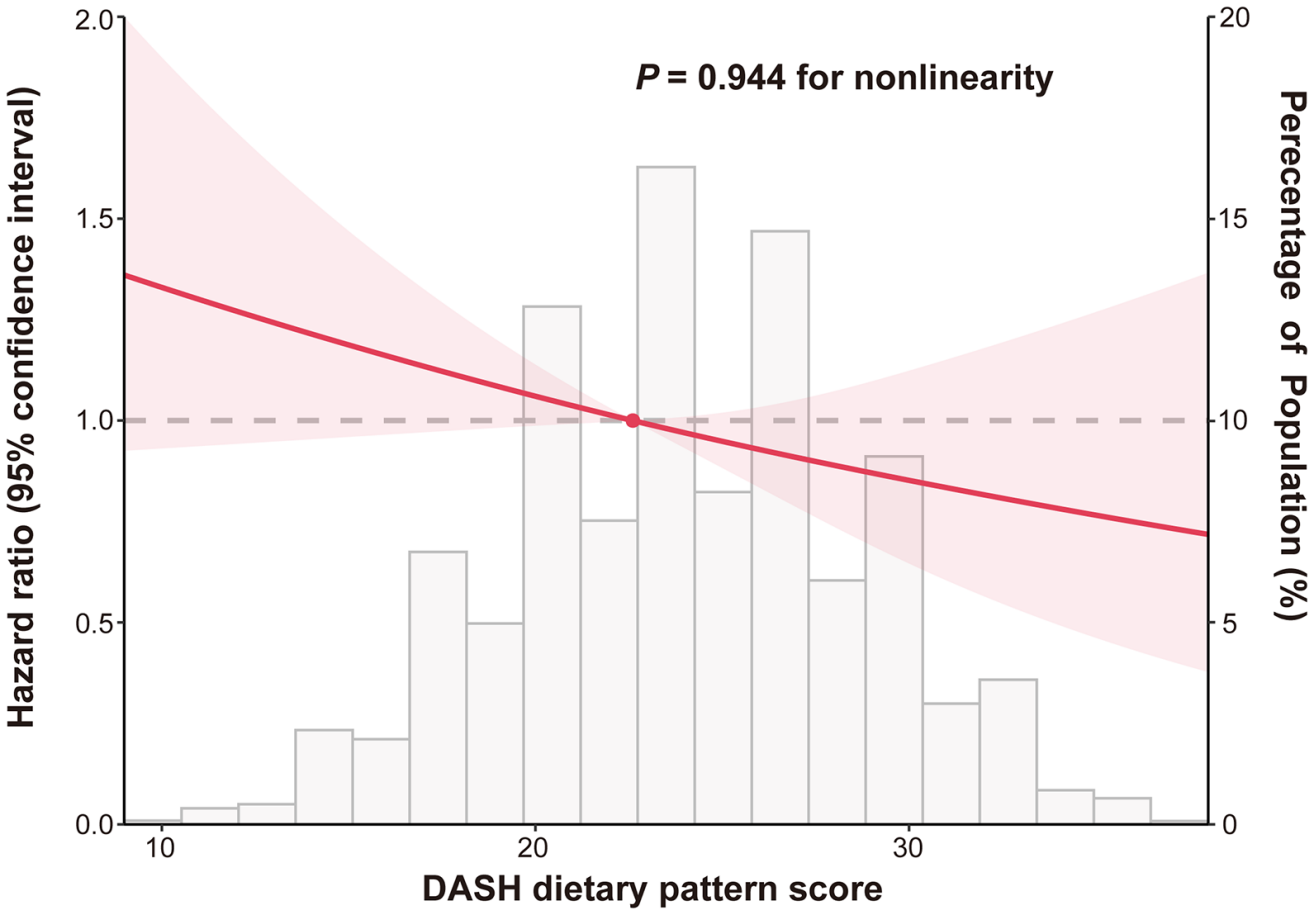
Dose–response association between DASH score and lung cancer risk. Hazard ratios were adjusted for age (years), gender (male, female), race (white, non-white), drinking status (no, yes), smoking status (never, current/former), cigarettes smoked per day (0, 1–20, >20), pack-years (continuous), body mass index (continuous), randomization group (intervention group/control group), history of hypertension (no, yes), family history of lung cancer (no, yes/possible) and energy intake from diet (continuous).

In order to represent the risk of lung cancer across the complete range of DASH scores, a restricted cubic spline plot was used. As can be seen in Figure 3, the DASH score and the risk of lung cancer were inversely correlated and had a linear dose–response relationship (*P*_non-linear_ = 0.944). According to subgroup analyses, factors like age, gender, race, randomization group, BMI, drinking habits, family history of lung cancer, hypertension history, and dietary energy intake had no effect on the relationship between the DASH score and risk of developing lung cancer (*P*_interaction_ > 0.05). However, the inverse relationship was significantly stronger for participants who were current or former smokers than for never-smokers (*P*_interaction_ = 0.013) (Table 3). The inverse relationship between DASH score and lung cancer risk did not change significantly in sensitivity analysis after the individuals with missing data, those with a history of hypertension and a family history of lung cancer, and lung cancer cases assessed within the first two or four years of follow-up were eliminated, showing that the findings of this research have good stability (Table 4).

**Table 3:** Subgroup analyses on the association of DASH score with the risk of lung cancer.

Subgroup variable	No. of subjects	No. of cases	HR _Quartile 4 vs. Quartile 1_ (95% CI)^a^	*P*_*-*interaction_
**Age (years)**				0.140
≤65	28,099	370	0.764 (0.574, 1.018)	
>65	24,363	554	0.916 (0.754, 1.113)	
**Gender**				0.735
Male	26,037	566	0.902 (0.720, 1.128)	
Female	26,425	358	0.751 (0.597, 0.944)	
**Race**				0.098
White	48,378	865	0.808 (0.684, 0.954)	
Non-white	4,084	59	1.265 (0.709, 2.258)	
**Body mass index (kg/m**^**2**^**)**				0.914
≤25	18,172	366	0.839 (0.654, 1.078)	
>25	34,290	558	0.833 (0.676, 1.027)	
**Smoking status**				0.013
Never	24,588	61	1.202 (0.690, 2.093)	
Current/ Former	27,874	863	0.612 (0.520, 0.721)	
**Randomization group**				0.706
Intervention group	26,748	473	0.791 (0.630, 0.992)	
Control group	25,714	451	0.875 (0.698, 1.097)	
**Drinking status**				0.506
No	14,437	245	0.851 (0.631, 1.148)	
Yes	38,025	679	0.823 (0.681, 0.995)	
**Family history of lung cancer**				0.415
No	45,589	715	0.871 (0.727, 1.044)	
Yes/ Possible	6,873	209	0.707 (0.499, 1.001)	
**History of hypertension**				0.553
No	35,740	621	0.802 (0.658, 0.978)	
Yes	16,722	303	0.901 (0.687, 1.182)	
**Energy intake from diet (kcal/day)**				0.443
≤medium	26,233	442	0.803 (0.642, 1.004)	
>medium	26,229	482	0.861 (0.685, 1.081)	

a Adjusted for age (years), gender (male, female), race (white, non-white), drinking status (no, yes), smoking status (never, current/former), cigarettes smoked per day (0, 1–20, >20), pack-years (continuous), body mass index (continuous), randomization group (intervention group/control group), history of hypertension (no, yes), family history of lung cancer (no, yes/possible) and energy intake from diet (continuous).

**Table 4:** Sensitivity analyses on the association of DASH score with the risk of lung cancer.

Categories	HR _Quartile 4 vs. Quartile 1_ (95% confidence interval)^a^	*P*_-trend_
Repeated analysis in subjects with non-missing data Excluded subjects with a family history of lung cancer^b^	0.651 (0.558, 0.758) 0.671 (0.566, 0.794)	<0.001 <0.001
Excluded subjects with a history of hypertension^c^	0.614 (0.510, 0.739)	<0.001
Excluded cases observed within the first 2 years of follow-up	0.613 (0.519, 0.725)	<0.001
Excluded cases observed within the first 4 years of follow-up	0.586 (0.483, 0.711)	<0.001

a Adjusted for age (years), gender (male, female), race (white, non-white), drinking status (no, yes), smoking status (never, current/former), cigarettes smoked per day (0, 1–20, >20), pack-years (continuous), body mass index (continuous), randomization group (intervention group/control group), history of hypertension (no, yes), family history of lung cancer (no, yes/possible) and energy intake from diet (continuous).

b HRs was not adjusted for history of lung cancer.

c HRs was not adjusted for history of hypertension.

### Individual components and lung cancer risk

The link between the intake of all eight components of the DASH pattern and lung cancer risk was investigated. Higher fruit consumption was linked to a lower risk of lung cancer, according to Supplementary Table 3 (HR_quartile 4 versus 1_: 0.758; 95% CI: 0.657,0.873; *P*_trend_ < 0.001), this inverse relationship was also found for vegetables, nuts and legumes, whole grains, and low-fat dairy products. For red and processed meats, increased intake indicated the increased possibility of developing lung cancer (HR_quartile 4 versus 1_: 1.409; 95% CI: 1.203,1.650; *P*_trend_ < 0.001). However, for sodium and sweetened beverages, higher consumption was linked to a decreased risk. (For sodium, HR_quartiles 4 versus 1_: 0.694; 95% CI: 0.553,0.870; *P*_trend_ = 0.002; For sweetened beverages, HR _quartile 4 versus 1_: 0.795; 95% CI: 0.691,0.914; *P*_trend_ = 0.007).

## Discussion

It was discovered in the current study that adherence to the DASH eating pattern was linked to a lower risk of lung cancer (small cell carcinoma as well as non-small cell carcinoma) in accordance with the prospective data from the PLCO trial. The restricted cubic spline plot showed that the inverse relationship between lung cancer risk and DASH score followed a linear dose–response relationship. Subgroup analyses revealed that DASH eating pattern was more protective against lung cancer among current or former smokers. Furthermore, the results remained stable after excluding participants who might have influenced the results, which strengthens the conclusions.

DASH dietary pattern was originally proposed as a dietary model for preventing and controlling hypertension and has shown significant advantages in blood pressure control and metabolic diseases (21). However, as this dietary pattern becomes better understood, it has been linked to a number of cancers. For instance, participants in case–control research with 1,050 Iranian women revealed that those with the highest quartile of the DASH score had 85% reduced risk of developing breast cancer than those with the lowest quartile (22). According to another hospital-based case–control study that included 454 participants (178 histo-pathologically confirmed gastric cancer patients and 276 matched healthy controls), the highest adherence to the DASH dietary pattern was linked to a 54% lower risk of gastric cancer after adjusting for relevant confounders (23). Additionally, a meta-analysis conducted in 2020 revealed that following the DASH dietary pattern was related to a lowered risk of colorectal cancer (24). To our knowledge, Myneni et al. has investigated the association between DASH dietary pattern and lung cancer risk in a population of 86,090 perimenopausal women (25), and their results showed that adherence to the DASH diet was not linked to an overall risk of developing lung cancer but reduced the risk of squamous cell carcinoma by up to 13%. In addition, Anic et al. investigated the correlation between four diet quality indices and lung cancer risk, and found that the DASH diet pattern had a 16% reduced risk of lung cancer (26). Compared with the previous studies, our results suggest DASH was protective against lung cancer and its subtypes. One potential explanation for the inconsistent results mentioned above is that Myneni et al.’s study only included female participants, whereas our research involved a population with a nearly equal distribution of both males and females. Additionally, there were differences in the covariates included in previous and our studies. Although smoking factors were comprehensively adjusted in previous studies, family history of lung cancer and hypertension history were not considered. Individuals with a higher score of DASH diet would be less likely to have a history of hypertension (27), and individuals with a family history of lung cancer may be at an increased risk of developing lung cancer (28). However, we adequately accounted for these crucial factors in our multivariable model.

Based on our findings of the relationship between the consumption of all eight components and lung cancer risk, it was found that adding adequate amounts of fruits, vegetables, nuts and legumes, whole grains, and low-fat dairy products was linked to reduced lung cancer risk, higher intake of red and processed meat increased the risk of developing lung cancer. The association of these dietary components with lung cancer risk was largely supported by former studies (7, 29), and further supporting the potential rationale for the inverse association between the DASH diet and the risk of developing lung cancer. Interestingly, dietary sodium and sugar-sweetened beverages, which are advocated to reduce consumption in the DASH diet, may have potential protective effects against lung cancer as presented in our study. These results were inconsistent with previous evidence that higher intake of sugar-sweetened beverages and dietary sodium were associated with an increased risk of various cancers (8, 30). Although direct evidence linking sugar-sweetened beverages to lung cancer risk is currently lacking, they are believed to be closely associated with risk factors for lung cancer, such as insulin resistance, inflammation, obesity, and type 2 diabetes (31). Further basic research and comprehensive epidemiological studies are needed to clarify the relationship between sugar-sweetened beverages and lung cancer. In addition, our study found no significant association between dietary sodium intake and lung cancer risk in both the unadjusted model and adjusted model 1. However, in the fully adjusted model 2, higher dietary sodium intake was associated with a lower risk of lung cancer. It is possible that this finding was incidental due to interactions between dietary sodium intake and the covariates considered in our study. On the other hand, research focused solely on individual nutrients or foods has not adequately explored the complex interplay between dietary components (32).

The benefits of the DASH diet in lowering the risk of developing lung cancer might be attributed to multiple underlying mechanisms. Firstly, fruits, vegetables, and grains are rich in phytochemicals with antioxidant activity, such as β-carotene and vitamin C (33). Evidence shows that oxidative stress can cause intracellular DNA base changes, strand breaks, overexpression of proto-oncogenes, and inactivation of oncogenes, resulting in the growth of certain malignancies, including lung cancer (34–36). Adherence to the DASH diet may lower the risk of lung cancer by increasing antioxidant capacity. Secondly, the DASH diet requires a limited consumption of red and processed meats, which are rich in many carcinogens such as aromatic amines and nitrites, and these substances can promote cancer development by causing DNA damage (37, 38). Thirdly, persistent inflammation-induced generation of reactive oxygen/nitrogen species in the lungs may increase the risk of lung cancer (39, 40). Animal studies have demonstrated that eating large amounts of fiber, which is abundant in fruits, vegetables and grains, can change the composition of lung microbiota, which in turn remodels the immune environment of the lungs (41). Moreover, previous studies have shown the benefits of adhering to the DASH diet in improving circulating serum inflammatory biomarkers such as highly sensitive C-reactive protein, which suggested that DASH diet might be able to reduce the inflammatory response of the body (42). Fourthly, some components, such as low-fat dairy products, may reduce the insulin resistance (43), which was demonstrated to be closely related to increased risk of lung cancer (44, 45). To sum up, adherence to DASH diet may potentially reduce lung cancer risk through mechanisms that involve increased antioxidant capacity, anti-inflammatory responses, and improved insulin resistance. Nevertheless, more research is necessary to confirm these mechanisms.

In subgroup analyses, it was found that for current or former smokers, there were more benefits from adherence to the DASH diet in terms of lung cancer prevention compared to those who never smoked. One possible explanation is that only a few cases of lung cancer were observed in individuals who never smoked, causing the loss of sufficient statistical efficiency. Another possible rationale is that oxidative stress and inflammation in the lung are alleviated by the DASH diet, while a vital mechanism of smoking-induced lung cancer is that smoking-related oxidative stress causes inflammation and potentially increased oxidative damage to lung tissue (46). Regardless, it was suggested in the findings of this research that adherence to the DASH diet may be more meaningful for current or former smokers. The underlying mechanisms need to be confirmed by further studies.

This study has distinct advantages. (i) This study was a well-designed prospective study in a large population and up to 8.84 years follow-up period ensured that the outcome events could occur; (ii) Consistent findings were obtained by analyzing the association between the DASH dietary pattern and each component and lung cancer risk, which increased the credibility of the conclusion. (iii) Our study provided dietary guidance to the US population in terms of lung cancer prevention, especially for current or former smokers.

However, there are several limitations to our study. (i) Participants included were the US population aged 55–74 years, the findings cannot be extended to other regions or ages; (ii) Although food frequency questionnaire is a well-designed dietary evaluation tool (17), the fact that participants self-report dietary history information still introduced bias into our study. The bias was non-differential and often unavoidable in epidemiological investigations. (iii) Collecting dietary information at once without considering possible changes in the dietary habits of individuals during the follow-up period might lead to non-differential bias. However, current nutritional epidemiology research suggests that the eating habits of individuals do not usually alter dramatically (20). Secondly, using one-time baseline data for cancer risk analysis tends to yield a weaker association than using cumulative average food consumption over a period of time (47); (iv) Although most potential confounders were adjusted in model 2, there are still some possible risk factors for lung cancer that were not excluded, such as passive smoking (48, 49) and air pollution (5), these factors cannot be further adjusted due to the unavailability of the data. However, participants included in our study were derived from ten centers across the United States, which could partially eliminate the effect of air pollution on the incidence rate of lung cancer.

## Conclusion

In the US population, DASH scores were inversely linked to the risk of developing lung cancer. This suggests that adherence to the DASH dietary pattern can be beneficial in lung cancer prevention, especially for current or former smokers. More epidemiological evidence from other regions and populations is needed to confirm and strengthen the findings of this research.

## Data availability statement

The raw data supporting the conclusions of this article will be made available by the authors, without undue reservation.

## Ethics statement

The studies involving human participants were reviewed and approved by Institutional Review Board of the National Cancer Institute. The patients/participants provided their written informed consent to participate in this study.

## Author contributions

ZZ, LX, and YW designed the study. LP and LX collected the raw data. ZZ, HG, and YX analyzed the data. YT, MY, and HH assisted in the interpretation of the results. ZZ and LX drafted the manuscript, YW reviewed the manuscript and finalized it. All authors contributed to the article and approved the submitted version.

## Funding

This work was funded by the General Project of Chongqing Natural Science Foundation, Chongqing Science and Technology Commission, China (cstc2021jcyj-msxmX0112, CSTB2022NSCQ-MSX1005, and cstc2021jcyj-msxmX0153).

## Conflict of interest

The authors declare that the research was conducted in the absence of any commercial or financial relationships that could be construed as a potential conflict of interest.

## Publisher’s note

All claims expressed in this article are solely those of the authors and do not necessarily represent those of their affiliated organizations, or those of the publisher, the editors and the reviewers. Any product that may be evaluated in this article, or claim that may be made by its manufacturer, is not guaranteed or endorsed by the publisher.
