# Supplementary material for: Association between dietary approaches to stop hypertension eating pattern and lung cancer risk in 98,459 participants: results from a large prospective study
Source: Front Nutr. 2023 May 15;10:1142067. doi: 10.3389/fnut.2023.1142067 (PMC10225695; doi:10.3389/fnut.2023.1142067)
Supplement: Supplementary file 1 [file Table_1.docx]

| **Supplemental Table 1. Criteria for determining DASH score** | | | | | | | | | |
| --- | --- | --- | --- | --- | --- | --- | --- | --- | --- |
| **Energy-adjusted dietary intakes of individual components** | | | | | | | | | |
| **Points** | **Fruits (g/day)** | **Nuts and legumes(g/day)** | | **Vegetables (g/day)** | **Grains**  **(g/day)** | **Low-fat dairy(g/day)** | **Dietary sodium (mg/day)** | **Red/processed meat(g/day)** | **Sugar-sweetened beverage(g/day)** |
| 5 | ≥398.38 | | ≥29.07 | ≥395.06 | ≥91.88 | ≥216.43 | ≤1781.35 | ≤2.74 | ≤104.33 |
| 4 | 278.59–398.37 | | 16.51–29.06 | 283.25–395.05 | 56.69–91.87 | 74.66–216.42 | 1781.36–2281.25 | 2.75–5.57 | 104.34–210.73 |
| 3 | 190.73–278.58 | | 10.19–16.50 | 210.55–283.24 | 36.29–56.68 | 20.99–74.65 | 2281.26–2803.94 | 5.58–9.92 | 210.74–331.97 |
| 2 | 110.52–190.72 | | 5.40–10.18 | 145.24–210.54 | 17.77–36.28 | 5.75–20.98 | 2803.95–3560.78 | 9.93–18.74 | 331.98–576.52 |
| 1 | ≤110.51 | ≤5.39 | | ≤145.23 | ≤17.76 | ≤5.74 | ≥3560.79 | ≥18.75 | ≥576.53 |
