# Supplementary material for: Association between dietary approaches to stop hypertension eating pattern and lung cancer risk in 98,459 participants: results from a large prospective study
Source: Front Nutr. 2023 May 15;10:1142067. doi: 10.3389/fnut.2023.1142067 (PMC10225695; doi:10.3389/fnut.2023.1142067)
Supplement: Supplementary file 2 [file Table_2.DOCX]

**Supplemental Table 2. Distribution of covariates with missing data before and after imputation^a^**

| **Variable** | **Before imputation** | **After imputation** **^a^** | **Number (%) with missing data** |
| --- | --- | --- | --- |
| **Family history of lung cancer** |  |  | 757 (0.77%) |
| No | 85088 (87.09%) | 85845 (87.19%) |  |
| Yes | 10266 (10.51%) | 10266 (10.43%) |  |
| Possible | 2348 (2.40%) | 2348 (2.38%) |  |
| **Race** |  |  | 34 (0.03%) |
| White | 91187(92.65%) | 91221(92.65%) |  |
| Non-White | 7238(7.35%) | 7238(7.35%) |  |
| **Body mass index (kg/m^2^)** | 27.21±4.82 | 27.20±4.79 | 1293 (1.31%) |
| **Smoking status** |  |  | 20 (0.02%) |
| Never | 47213 (47.96%) | 47233 (47.97%) |  |
| Current | 8993 (9.14%) | 8993 (9.14%) |  |
| Former | 42233 (42.90%) | 42233 (42.89%) |  |
| **Cigarettes smoked per day** |  |  | 120 (0.12%) |
| 0 | 47213(48.01%) | 47233(47.97%) |  |
| 1-20 | 32097(32.64%) | 32197(32.70%) |  |
| >20 | 19029(19.35%) | 19029(19.33%) |  |
| **Pack-years** | 17.67±26.50 | 17.49±26.40 | 1105 (1.12%) |
| **History of hypertension** |  |  | 494 (0.50%) |
| No | 66147 (67.52%) | 66641 (67.68%) |  |
| Yes | 31818 (32.48%) | 31818 (32.32%) |  |

^a^ Values are mean (standard deviation) or counts (percentage) as indicated.
