# Supplementary material for: Association between dietary approaches to stop hypertension eating pattern and lung cancer risk in 98,459 participants: results from a large prospective study
Source: Front Nutr. 2023 May 15;10:1142067. doi: 10.3389/fnut.2023.1142067 (PMC10225695; doi:10.3389/fnut.2023.1142067)
Supplement: Supplementary file 3 [file Table_3.docx]

**Supplementary Table3.1 Hazard ratios of the association of fruits with the risk of lung cancer**

| **Quartiles of fruit intake (g/day)** | **Number of subjects/cases** | | **Person-years** | **Incidence rate per 100 person-years (95% confidence interval)** | **Hazard ratio (95% confidence interval)** | | | |
| --- | --- | --- | --- | --- | --- | --- | --- | --- |
|  |  |  |  |  | **Unadjusted** | **Model 1^a^** | **Model 2^b^** | |
| Quartile 1 (≤131.71) | 24616/562 | | 213492.8 | 0.263 (0.242, 0.286) | 1.000 (reference) | 1.000 (reference) | | 1.000 (reference) |
| Quartile 2 (131.72-234.24) | | 24614/412 | 217248.7 | 0.190 (0.172, 0.209) | 0.718 (0.632,0.815) | 0.677 (0.596,0.769) | | 0.856 (0.753,0.973) |
| Quartile 3 (234.25-361.46) | | 24614/337 | 219052.2 | 0.154 (0.138, 0.171) | 0.582 (0.508,0.666) | 0.538 (0.469,0.616) | | 0.752 (0.655,0.863) |
| Quartile 4 (>361.46) | 24615/331 | | 220014.3 | 0.150 (0.135, 0.168) | 0.568 (0.496,0.651) | 0.519 (0.453,0.595) | | 0.758 (0.657,0.873) |
| *P* for trend |  | |  |  | <0.001 | <0.001 | | <0.001 |

**^a^** Adjusted for age (years), gender (male, female) and race (white, non-white).

**^b^** Adjusted for model 1 plus drinking status (no, yes), smoking status (never, current/ former), cigarettes smoked per day (0, 1-20, >20), number of packs smoked per day * years smoked (continuous), body mass index (continuous), randomization group (intervention group/ control group), history of hypertension (no, yes), family history of lung cancer (no, yes/ possible) and energy intake from diet (continuous).

**Supplementary Table3.2 Hazard ratios of the association of low-fat dairy products with the risk of lung cancer**

| **Quartiles of low-fat dairy intake (g/day)** | **Number of subjects/cases** | **Person-years** | **Incidence rate per 100 person-years (95% confidence interval)** | **Hazard ratio (95% confidence interval)** | | | |
| --- | --- | --- | --- | --- | --- | --- | --- |
|  |  |  |  | **Unadjusted** | **Model 1^a^** | **Model 2^b^** | |
| Quartile 1 (≤8.88) | 24736/585 | 214992.5 | 0.272 (0.251, 0.295) | 1.000 (reference) | 1.000 (reference) | | 1.000 (reference) |
| Quartile 2 (8.89-40.03) | 24612/411 | 216421.0 | 0.190 (0.172, 0.209) | 0.697 (0.614,0.791) | 0.706 (0.622,0.802) | | 0.814 (0.716,0.924) |
| Quartile 3 (40.04-165.52) | 24496/339 | 217747.6 | 0.156 (0.140, 0.173) | 0.571 (0.499,0.652) | 0.599 (0.523,0.685) | | 0.715 (0.624,0.819) |
| Quartile 4 (>165.52) | 24615/307 | 220646.7 | 0.139 (0.124, 0.156) | 0.509 (0.444,0.585) | 0.517 (0.450,0.595) | | 0.663 (0.575,0.763) |
| *P* for trend |  |  |  | <0.001 | <0.001 | | <0.001 |

**^a^** Adjusted for age (years), gender (male, female) and race (white, non-white).

**^b^** Adjusted for model 1 plus drinking status (no, yes), smoking status (never, current/ former), cigarettes smoked per day (0, 1-20, >20), number of packs smoked per day * years smoked (continuous), body mass index (continuous), randomization group (intervention group/ control group), history of hypertension (no, yes), family history of lung cancer (no, yes/ possible) and energy intake from diet (continuous).

**Supplementary Table3.3 Hazard ratios of the association of vegetables with the risk of lung cancer**

| **Quartiles of vegetables intake (g/day)** | **Number of subjects/cases** | **Person-years** | **Incidence rate per 100 person-years (95% confidence interval)** | **Hazard ratio (95% confidence interval)** | | | |
| --- | --- | --- | --- | --- | --- | --- | --- |
|  |  |  |  | **Unadjusted** | **Model 1^a^** | **Model 2^b^** | |
| Quartile 1 (≤ 162.51) | 24615/460 | 214969.9 | 0.214 (0.195, 0.234) | 1.000 (reference) | 1.000 (reference) | | 1.000 (reference) |
| Quartile 2 (162.52-245.06) | 24617/416 | 218081.3 | 0.191 (0.173, 0.210) | 0.890 (0.779,1.016) | 0.886 (0.776,1.012) | | 0.962 (0.841,1.100) |
| Quartile 3 (245.07-359.76) | 24614/389 | 217866.2 | 0.179 (0.162, 0.197) | 0.833 (0.728,0.953) | 0.828 (0.723,0.948) | | 0.884 (0.768,1.017) |
| Quartile 4 (>359.76) | 24613/377 | 218890.5 | 0.172 (0.156, 0.190) | 0.803 (0.700,0.920) | 0.788 (0.687,0.903) | | 0.827 (0.711,0.963) |
| *P* for trend |  |  |  | 0.002 | <0.001 | | 0.009 |

**^a^** Adjusted for age (years), gender (male, female) and race (white, non-white).

**^b^** Adjusted for model 1 plus drinking status (no, yes), smoking status (never, current/ former), cigarettes smoked per day (0, 1-20, >20), number of packs smoked per day * years smoked (continuous), body mass index (continuous), randomization group (intervention group/ control group), history of hypertension (no, yes), family history of lung cancer (no, yes/ possible) and energy intake from diet (continuous).

**Supplementary Table3.4 Hazard ratios of the association of whole grains with the risk of lung cancer**

| **Quartiles of whole grains intake (g/day)** | **Number of subjects/cases** | **Person-years** | **Incidence rate per 100 person-years (95% confidence interval)** | **Hazard ratio (95% confidence interval)** | | | |
| --- | --- | --- | --- | --- | --- | --- | --- |
|  |  |  |  | **Unadjusted** | **Model 1^a^** | **Model 2^b^** | |
| Quartile 1 (≤ 22.20) | 24642/511 | 215723.5 | 0.237 (0.217, 0.258) | 1.000 (reference) | 1.000 (reference) | | 1.000 (reference) |
| Quartile 2 (22.21-46.12) | 24595/414 | 217974.5 | 0.190 (0.173, 0.209) | 0.800 (0.703,0.911) | 0.763 (0.670,0.868) | | 0.943 (0.827,1.074) |
| Quartile 3 (46.13-80.50) | 24607/371 | 218602.8 | 0.170 (0.153, 0.188) | 0.715 (0.626,0.817) | 0.643 (0.562,0.735) | | 0.847 (0.739,0.970) |
| Quartile 4 (>80.50) | 24615/346 | 217507.1 | 0.159 (0.143, 0.177) | 0.670 (0.585,0.768) | 0.579 (0.505,0.665) | | 0.800 (0.694,0.921) |
| *P* for trend |  |  |  | <0.001 | <0.001 | | 0.001 |

**^a^** Adjusted for age (years), gender (male, female) and race (white, non-white).

**^b^** Adjusted for model 1 plus drinking status (no, yes), smoking status (never, current/ former), cigarettes smoked per day (0, 1-20, >20), number of packs smoked per day * years smoked (continuous), body mass index (continuous), randomization group (intervention group/ control group), history of hypertension (no, yes), family history of lung cancer (no, yes/ possible) and energy intake from diet (continuous).

**Supplementary Table3.5 Hazard ratios of the association of nuts and legumes with the risk of lung cancer**

| **Quartiles of nuts intake (g/day)** | **Number of subjects/cases** | **Person-years** | **Incidence rate per 100 person-years (95% confidence interval)** | **Hazard ratio (95% confidence interval)** | | | |
| --- | --- | --- | --- | --- | --- | --- | --- |
|  |  |  |  | **Unadjusted** | **Model 1^a^** | **Model 2^b^** | |
| Quartile 1 (≤6.61) | 24630/481 | 216125.4 | 0.223 (0.204, 0.243) | 1.000 (reference) | 1.000 (reference) | | 1.000 (reference) |
| Quartile 2 (6.62-13.13) | 20606/402 | 217758.7 | 0.185 (0.167, 0.204) | 0.829 (0.726,0.946) | 0.806 (0.706,0.920) | | 0.889 (0.778,1.015) |
| Quartile 3 (13.14-25.39) | 24620/372 | 217968.2 | 0.171 (0.154, 0.189) | 0.766 (0.669,0.877) | 0.730 (0.638,0.836) | | 0.834 (0.726,0.957) |
| Quartile 4 (>25.39) | 24603/387 | 217955.6 | 0.178 (0.161, 0.196) | 0.797 (0.697,0.911) | 0.751 (0.656,0.859) | | 0.828 (0.718,0.956) |
| *P* for trend |  |  |  | 0.007 | <0.001 | | 0.025 |

**^a^** Adjusted for age (years), gender (male, female) and race (white, non-white).

**^b^** Adjusted for model 1 plus drinking status (no, yes), smoking status (never, current/ former), cigarettes smoked per day (0, 1-20, >20), number of packs smoked per day * years smoked (continuous), body mass index (continuous), randomization group (intervention group/ control group), history of hypertension (no, yes), family history of lung cancer (no, yes/ possible) and energy intake from diet (continuous).

**Supplementary Table3.6 Hazard ratios of the association of sodium with the risk of lung cancer**

| **Quartiles of sodium intake (mg/day)** | **Number of subjects/cases** | **Person-years** | **Incidence rate per 100 person-years (95% confidence interval)** | **Hazard ratio (95% confidence interval)** | | | |
| --- | --- | --- | --- | --- | --- | --- | --- |
|  |  |  |  | **Unadjusted** | **Model 1^a^** | **Model 2^b^** | |
| Quartile 1 (≤1910.68) | 24615/408 | 216709.7 | 0.188 (0.171, 0.207) | 1.000 (reference) | 1.000 (reference) | | 1.000 (reference) |
| Quartile 2 (1910.69-2530.01) | 24615/395 | 217845.5 | 0.181 (0.164, 0.200) | 0.962 (0.838,1.105) | 0.927 (0.807,1.065) | | 0.879 (0.759,1.017) |
| Quartile 3 (2530.02-3326.56) | 24614/417 | 218109.6 | 0.191 (0.174, 0.210) | 1.014 (0.885,1.163) | 0.942 (0.820,1.083) | | 0.832 (0.705,0.980) |
| Quartile 4 (>3326.56) | 24615/422 | 217143.0 | 0.194 (0.177, 0.214) | 1.031 (0.900,1.182) | 0.921 (0.798,1.062) | | 0.694 (0.553,0.870) |
| *P* for trend |  |  |  | 0.486 | 0.348 | | 0.002 |

**^a^** Adjusted for age (years), gender (male, female) and race (white, non-white).

**^b^** Adjusted for model 1 plus drinking status (no, yes), smoking status (never, current/ former), cigarettes smoked per day (0, 1-20, >20), number of packs smoked per day * years smoked (continuous), body mass index (continuous), randomization group (intervention group/ control group), history of hypertension (no, yes), family history of lung cancer (no, yes/ possible) and energy intake from diet (continuous).

**Supplementary Table3.7 Hazard ratios of the association of sweetened beverages with the risk of lung cancer**

| **Quartiles of sweetened beverages intake (g/day)** | **Number of subjects/cases** | **Person-years** | **Incidence rate per 100 person-years (95% confidence interval)** | **Hazard ratio (95% confidence interval)** | | | |
| --- | --- | --- | --- | --- | --- | --- | --- |
|  |  |  |  | **Unadjusted** | **Model 1^a^** | **Model 2^b^** | |
| Quartile 1 (≤130.55) | 24615/524 | 216951.9 | 0.242 (0.222, 0.263) | 1.000 (reference) | 1.000 (reference) | | 1.000 (reference) |
| Quartile 2 (130.56-264.87) | 24615/379 | 218417.0 | 0.174 (0.157, 0.192) | 0.717 (0.629,0.819) | 0.703 (0.616,0.802) | | 0.830 (0.726,0.947) |
| Quartile 3 (264.88-495.22) | 24614/380 | 218026.8 | 0.174 (0.158, 0.193) | 0.721 (0.632,0.823) | 0.717 (0.628,0.818) | | 0.876 (0.766,1.001) |
| Quartile 4 (>495.22) | 24615/359 | 216412.2 | 0.166 (0.150, 0.184) | 0.687 (0.600,0.785) | 0.719 (0.628,0.824) | | 0.795 (0.691,0.914) |
| *P* for trend |  |  |  | <0.001 | <0.001 | | 0.007 |

**^a^** Adjusted for age (years), gender (male, female) and race (white, non-white).

**^b^** Adjusted for model 1 plus drinking status (no, yes), smoking status (never, current/ former), cigarettes smoked per day (0, 1-20, >20), number of packs smoked per day * years smoked (continuous), body mass index (continuous), randomization group (intervention group/ control group), history of hypertension (no, yes), family history of lung cancer (no, yes/ possible) and energy intake from diet (continuous).

**Supplementary Table3.8 Hazard ratios of the association of red and processed meats with the risk of lung cancer**

| **Quartiles of red/processed meat intake (g/day)** | **Number of subjects/cases** | **Person-years** | **Incidence rate per 100 person-years (95% confidence interval)** | **Hazard ratio (95% confidence interval)** | | | |
| --- | --- | --- | --- | --- | --- | --- | --- |
|  |  |  |  | **Unadjusted** | **Model 1^a^** | **Model 2^b^** | |
| Quartile 1 (≤3.38) | 24619/317 | 220069.2 | 0.144 (0.129, 0.161) | 1.000 (reference) | 1.000 (reference) | | 1.000 (reference) |
| Quartile 2 (3.39-7.48) | 24625/352 | 218445.4 | 0.161 (0.145, 0.179) | 1.120 (0.962,1.304) | 1.108 (0.951,1.291) | | 1.079 (0.926,1.258) |
| Quartile 3 (7.49-15.74) | 24601/425 | 216552.1 | 0.196 (0.178, 0.216) | 1.365 (1.181,1.579) | 1.314 (1.132,1.525) | | 1.201 (1.032,1.399) |
| Quartile 4 (>15.74) | 24614/548 | 214741.1 | 0.255 (0.235, 0.277) | 1.777 (1.548,2.041) | 1.648 (1.423,1.908) | | 1.409 (1.203,1.650) |
| *P* for trend |  |  |  | <0.001 | <0.001 | | <0.001 |

**^a^** Adjusted for age (years), gender (male, female) and race (white, non-white).

**^b^** Adjusted for model 1 plus drinking status (no, yes), smoking status (never, current/ former), cigarettes smoked per day (0, 1-20, >20), number of packs smoked per day * years smoked (continuous), body mass index (continuous), randomization group (intervention group/ control group), history of hypertension (no, yes), family history of lung cancer (no, yes/ possible) and energy intake from diet (continuous).
